# Hepatoma-Derived Growth Factor Upregulation Is Correlated with Prognostic Factors of Early-Stage Cervical Adenocarcinoma

**DOI:** 10.3390/ijms151121492

**Published:** 2014-11-21

**Authors:** Ching-Chou Tsai, Shun-Chen Huang, Ming Hong Tai, Chan-Chao Chang Chien, Chao-Cheng Huang, Yi-Chiang Hsu

**Affiliations:** 1 Department of Obstetrics and Gynecology, Chang Gung Memorial Hospital, Kaohsiung and College of Medicine, Chang Gung University, Kaohsiung 83301, Taiwan; E-Mails: nick@cgmh.org.tw (C.-C.T.); chanchao@cgmh.org.tw (C.-C.C.C.); 2 Department of Pathology, Chang Gung Memorial Hospital, Kaohsiung and College of Medicine, Chang Gung University, Kaohsiung 83301, Taiwan; E-Mails: shuang@cgmh.org.tw (S.-C.H.); huangcc@cgmh.org.tw (C.-C.H.); 3 Department of Biological Sciences, National Sun Yat-Sen University, Kaohsiung 80424, Taiwan; E-Mail: minghongtai@gmail.com; 4 Graduate Institute of Medical Science, College of Health Sciences, Chang Jung Christian University, Tainan 71101, Taiwan; 5 Innovative Research Center of Medicine, College of Health Sciences, Chang Jung Christian University, Tainan 71101, Taiwan

**Keywords:** early-stage cervical adenocarcinoma (Cx), hepatoma-derived growth factor (HDGF), metastasis

## Abstract

Hepatoma-derived growth factor (HDGF) is a unique nuclear/growth factor that plays an important role in the progression of different types of cancer. A total of 63 patients with early-stage cervical adenocarcinoma (Cx) were enrolled in this retrospective study. The expression of HDGF was significantly increased compared with adjacent non-tumor tissue samples (*p <* 0.001). Moreover, elevated nuclear HDGF levels were correlated with lymph-vascular space invasion (LVSI; *p <* 0.05), lymph node metastasis (LNM; *p <* 0.001), recurrence (*p <* 0.001) and advanced grade (AG; *p <* 0.001). The growth of cervical cancer cells (Hela cells) was enhanced by HDGF treatment. The HDGF mRNA and protein level were significantly higher in malignant cervical cancer cells compared with primary ones. By adenovirus gene delivery, HDGF overexpression enhanced, whereas HDGF knockdown perturbed the tumorigenic behaviors of cervical cancer cells. HDGF overexpression is common in early-stage cervical adenocarcinoma and is involved in the carcinogenesis of cervical adenocarcinoma. Cytoplasmic HDGF expression is strongly correlated with pelvic lymph node metastasis and recurrence, indicating that HDGF may serve as a novel prognostic marker for patients with Cx.

## 1. Introduction

The prognosis of patients with cervical cancer is poor [1,2,3,4]. Combined surgical and chemo-radiotherapy treatment is needed in most cases, except for those with early disease. Radiotherapy is effective in selected patients; however, some patients suffer from side effects, such as immunosuppression [5]. Differentially-expressed genes between radio-sensitive and -resistant cancer cells have been studied, and one in particular, Hepatoma-derived growth factor (HDGF) has been identified, whose expression is suppressed in radio-resistant cell lines [6]. HDGF is a secreted growth factor that has been purified from the conditioned medium of human hepatoma and is an acidic polypeptide with heparin-binding growth stimulating activity for fibroblasts [7]. HDGF is ubiquitously expressed and is mitogenic for fibroblasts [8] and aortic endothelial cells [9]. HDGF is also highly expressed in several fetal tissues and may be involved in the development of organs, such as the lungs, muscles, vessels, kidneys and liver [10]. HDGF has a mitogenic function in various cells, such as human hepatocellular carcinoma cells, fibroblasts, endothelial cells, vascular smooth muscle cells and fetal hepatocytes [11]. Although the specific function of this protein is unknown, recent studies have suggested that HDGF plays a role in the regulation of cancer development [12]. Therefore, we hypothesized that the HDGF expression in human malignant tumors may also have an important functional role in metastasis and may consequently influence the prognosis of patients. In fact, several reports have found a correlation between an increased HDGF expression and poor prognosis in various cancers [13,14]. However, no reports have evaluated the correlation of HDGF expression with clinical pathological features and prognosis in cervical adenocarcinoma. In the present study, the expression levels of HDGF in 63 patients with cervical adenocarcinoma undergoing surgery and the relationship between the HDGF expression and clinical pathological features and prognosis were analyzed.

## 2. Results and Discussion

### 2.1. HDGF Expression and Location in the Tumor and Non-Tumor Specimens

**Figure 1:**
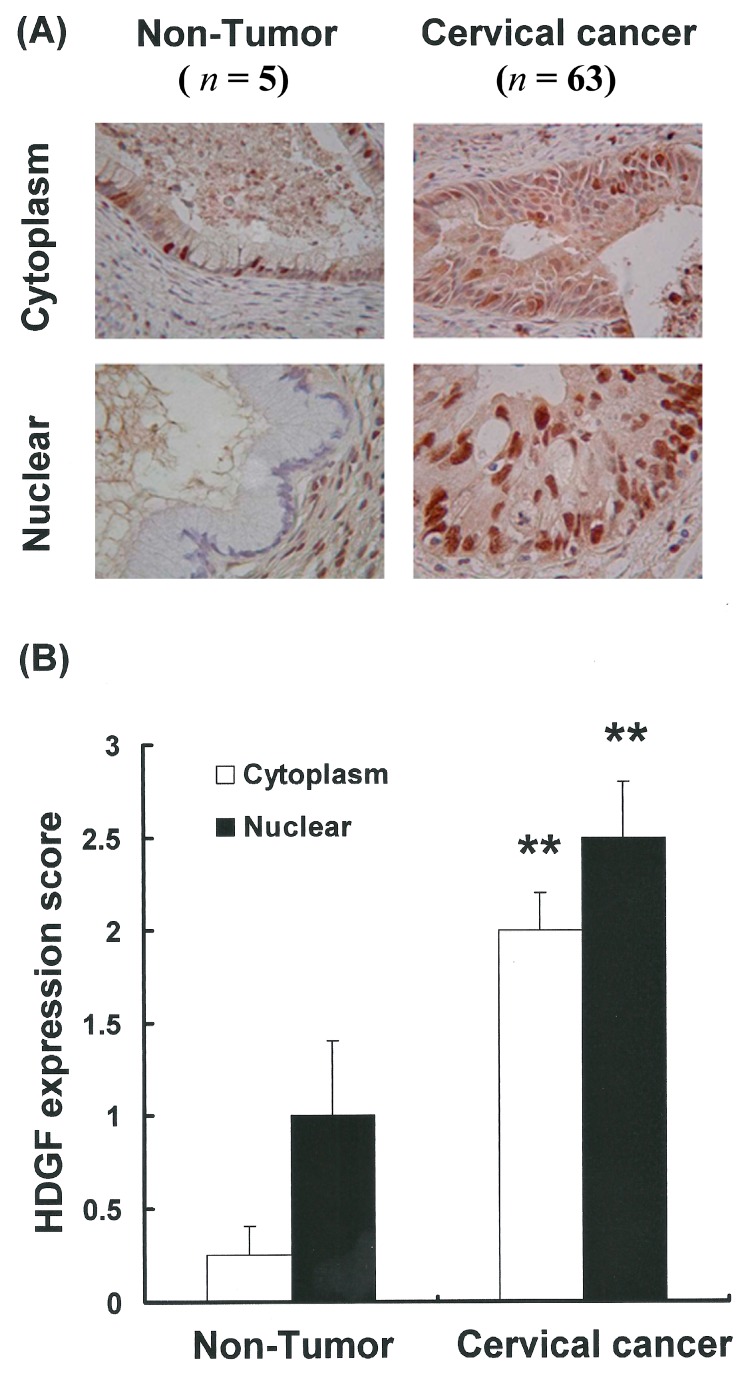
Differential nuclear and cytoplasm Hepatoma-derived growth factor (HDGF) expression in tumor and non-tumor specimens from patients with cervical adenocarcinoma (Cx). (**A**) HDGF expression in cervical cancer and non-tumor specimens from patients with Cx; (**B**) A higher percentage of HDGF-positive cells was found in the tumor regions than in the non-tumor regions. ** Indicates that the difference compared to the non-tumor samples was statistically significant at *p <* 0.01.

To further determine the extent and location of HDGF protein expression in cervical cancer tissues *in situ*, immunohistochemistry was performed using a monoclonal antibody specific for the HDGF protein. HDGF immunostaining was detected in the cervical cancer specimens (Figure 1A). Furthermore, the HDGF immunostaining was localized in both the cytoplasm and nuclei of the cells. Compared with adjacent non-tumor tissue samples, there was a statistically significant increase in nuclear HDGF expression in the cervical cancer tissue samples (** *p <* 0.001 *vs.* non-tumor; Figure 1B). Upregulation of HDGF was also observed in the cytoplasm of the cervical cancer tissue samples (*p <* 0.001 *vs.* non-tumor; Figure 1B). These results showed that the HDGF protein was expressed in the cervical cancer tissues and that increased HDGF levels may play an important role in cervical cancer.

### 2.2. Upregulation of HDGF Gene Expressions in Cervical Cancer Tissues

The western blot analysis revealed that the levels of HDGF protein expression were significantly increased (Figure S1A). In addition, western blot analysis also showed higher HDGF protein expressions in cervical cancer (Cx) tissues, but lower expressions in the non-tumor tissue controls (Figure S1A). Densitometry analysis showed a 9.695-fold overexpression of HDGF protein in seven Cx tissues compared to the non-tumor tissue controls (*p <* 0.01, Figure S1B). These results suggest that increased HDGF levels are present in poorly differentiated Cx cells.

### 2.3. Association between Nuclear and Cytoplasmic HDGF and Patient Survival

**Figure 2:**
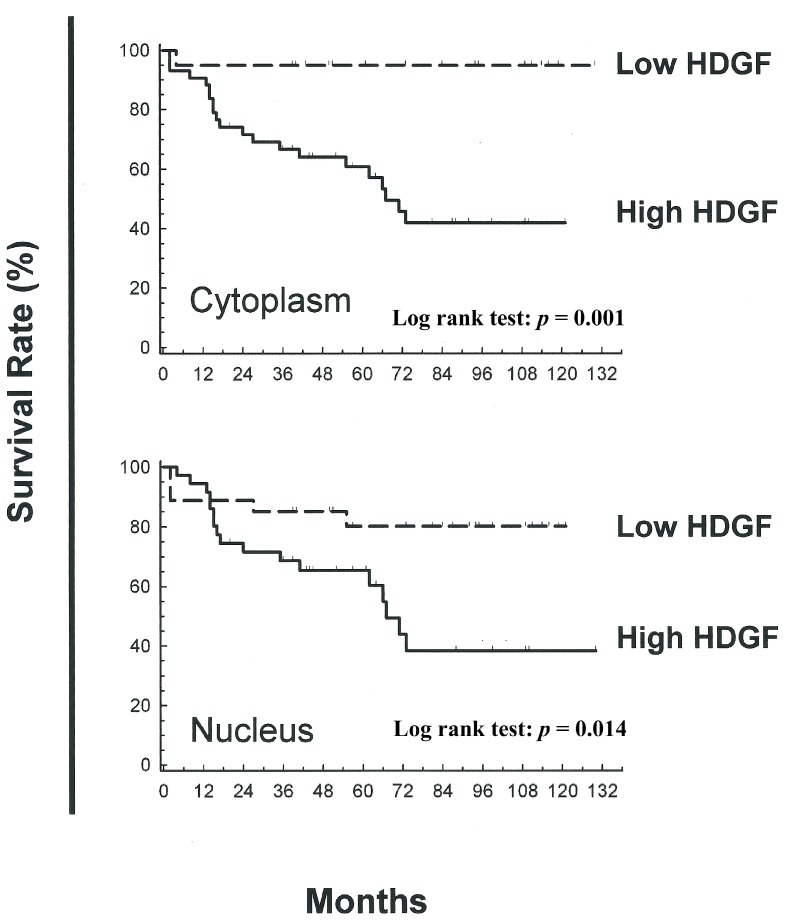
Kaplan-Meier analysis of overall survival rates for patients with cervical adenocarcinoma (Cx) exhibiting different subcellular HDGF expression levels. Correlation between cytoplasmic HDGF and overall survival. Patients with a lower expression of cytoplasmic HDGF had significantly more favorable survival rates compared with the patients with higher expression of cytoplasmic HDGF (*p* *=* 0.001). The correlation between nuclear HDGF and overall survival: patients with a lower expression of nuclear HDGF had better overall survival results than the patients with a higher expression of HDGF (*p* = 0.014).

Kaplan-Meier analysis revealed that the patients with Cx specimens exhibiting low nuclear and cytoplasmic expressions of HDGF had favorable overall survival results; the 1- to 120-month overall survival rates for these patients are shown in Figure 2. Furthermore, the patients with Cx specimens exhibiting low cytoplasmic expressions of HDGF also had a significantly longer disease-free survival. The 1-, 5- and 10-year overall survival rates were 90%, 80% and 80%, respectively, compared to 95%, 65% and 40%, respectively, for the patients with Cx specimens exhibiting high cytoplasmic HDGF (Figure 2). Similarly, the patients with Cx specimens exhibiting lower nuclear expressions of HDGF had better overall survival results.

### 2.4. Correlation of Increased HDGF Expression with Tumorigenicity of Cervical Cancer Cells

**Figure 3:**
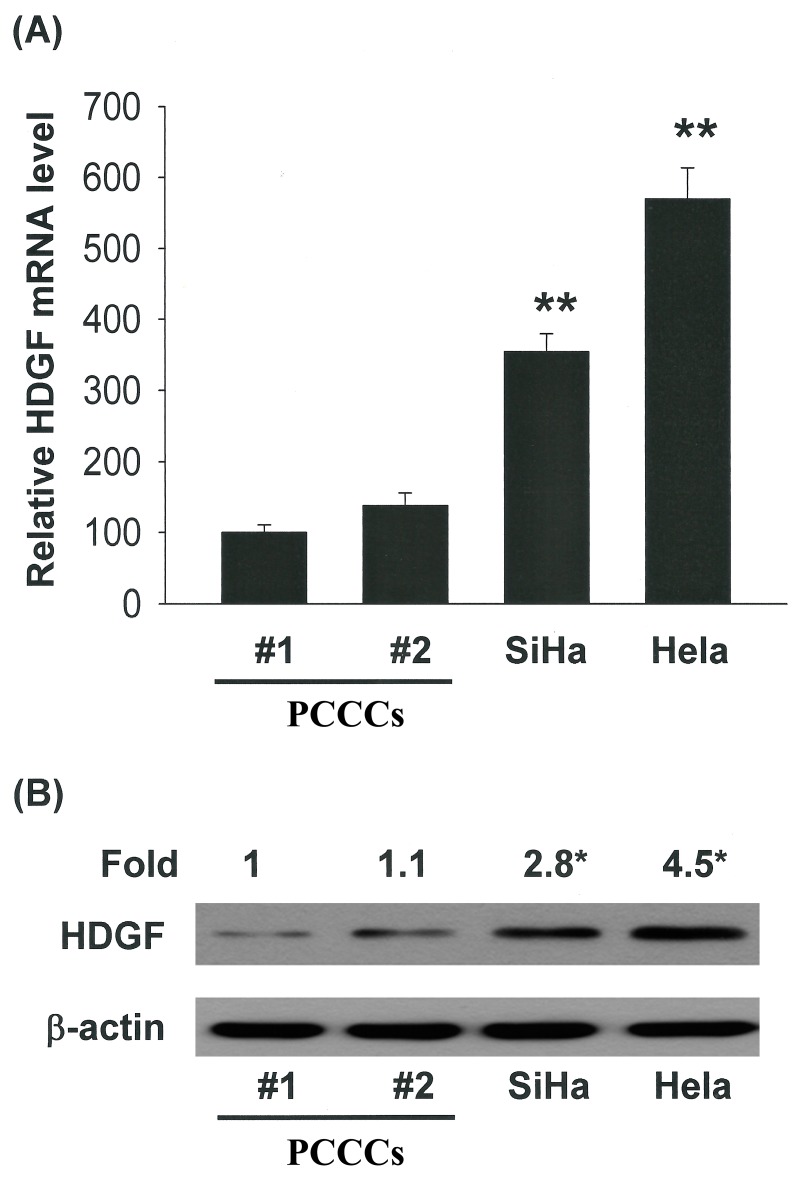
HDGF mediated the survival of cervical cancer cells, thereby increasing proliferation/growth. Correlation of HDGF expression with the tumorigenic behaviors of cervical cancer cells. (**A**) qRT-PCR and (**B**) immunoblot analysis of HDGF protein level in human cervical cell lines. All data are reported as the mean (±SEM) of at least three separate experiments. Statistical analysis was performed using a *t*-test, with significant differences determined at the level of * *p <* 0.05 and ** *p <* 0.01 *versus* the control group (HDGF 0 nM group).

To investigate the role of HDGF during cervical carcinogenesis, we examined HDGF expression in human cervical cell lines and found that the HDGF mRNA and protein level were significantly higher in malignant cervical cancer cells (SiHa and HeLa cells) than in benign ones (primary cervical cancer cells; PCCCs #1 and #2; Figure 3A,B, * *p <* 0.05 and ** *p <* 0.01).

### 2.5. HDGF Stimulated Cervical Cancer Cell Proliferation

HDGF is a known mitogen for endothelial cells in culture [15] and was found to be expressed in the Cx cells (Figure 1). Whether HDGF plays a role in regulating Cx growth is unknown [16,17,18]. To determine whether HDGF is a Cx mitogen, quiescent Cx cells (Hela cells) were treated with HDGF (0.02–0.2 nM). Cell viability was then measured 24 to 72 h after stimulation. As shown in Figure S2, HDGF stimulated a significant dose-dependent increase in cell number compared with the control group (HDGF 0 nM). At the lowest concentration used (0.02 nM), HDGF produced significant Cx proliferation (21% to 61%; *p <* 0.05). By using an invasion assay and colony formation assay (Figures S3 and S4), it was found that HeLa cells with the highest HDGF level also exhibited the most malignancy in invasiveness and anchorage-independent growth. Spearman’s correlation analysis further revealed a significant correlation of cellular HDGF level with the invasiveness and colony-forming capability of human cervical cancer cell lines (correlation coefficient: 0.92; *p <* 0.01). Thus, increased HDGF expression was associated with the tumorigenicity of cervical cancer cells.

### 2.6. The Cellular HDGF Level Regulates the Tumorigenicity of Cervical Cancer Cells

To validate the relationship between HDGF expression and the tumorigenicity of cervical cancer cells, we employed adenovirus gene delivery to modulate the cellular HDGF level, thereby investigating the influence of cellular HDGF level on tumorigenic behaviors in cervical cancer cells. Infection with adenovirus vectors encoding HDGF (Ad-HDGF) increased the HDGF protein level by about four-fold (Figure S5A) and led to a significant increment in the invasion and colony formation of HeLa cells (Figure S5B,C) (*p* < 0.01 *vs.* the primary control group; *p* < 0.01 *vs.* the Ad-HDGF group). Contrastingly, infection with adenovirus vectors encoding HDGF small-interfering RNA (Ad-HDGF RNAi) significantly decreased the HDGF protein level by more than 80% of the control and resulted in a significant reduction in the invasion and colony formation of HeLa cells. These data indicated that the cellular HDGF level regulated the tumorigenicity of cervical cancer cells.

HDGF overexpression appears to be an important event in cervical adenocarcinoma. The cytoplasmic HDGF expression may serve as a novel prognostic marker for patients with early-stage cervical adenocarcinoma.

Growth factors that may be involved in one or more steps in the development of cervical cancer include transforming growth factor-β [16], epidermal growth factor [13] and hepatocyte growth factor [17]. The novel growth factor HDGF has been shown to have high homology with high mobility group (HMG)-1 protein, and the protein and nucleotide sequence homologies, heparin binding capacity, molecular mass and ubiquitous expression suggest that HDGF might be a member of the HMG family [18]. Its cytoplasmic localization is due to an absence of the typical HMG box motif, suggesting that HDGF is a novel heparin-binding protein, which is distinct from HMG-1 in structural differences, the absence of the HMG box motif, the cytoplasmic localization and growth stimulating activity [19].

In the current study, we demonstrated that an increased HDGF expression was present in poorly differentiated Cx (Hela) cells and was associated with tumor progression [20,21,22,23]. Recently, inhibition of HDGF expression has been shown to repress the proliferation of hepatoma cells to varying degrees [12]. This is consistent with our findings of a differential HDGF expression in cervical adenocarcinoma tissues. Furthermore, altered HDGF levels may affect the proliferation and differentiation states of Cx cells.

How HDGF overexpression contributes to cervical adenocarcinoma carcinogenesis and influences the survival of patients with Cx remains to be elucidated. Because of the mitogenic activity of HDGF in Cx cells, an increased HDGF expression may enhance the unregulated growth or recurrence of Cx cells. In addition, we observed a positive correlation between HDGF immunostaining in the Cx specimens, which suggests that an elevated HDGF expression may stimulate angiogenic activity and promote tumor aggression. Together, these results provide a rational basis for oncogenic transformation due to HDGF upregulation. In the current study, the results of immunofluorescence and histological analysis clearly indicated that HDGF is localized in the nuclei and in the cytoplasm of Cx cells. Furthermore, the colocalization of HDGF with CD31 and the correlation between these two proteins supports the idea of the nuclear tropism of HDGF (data not shown). However, the cellular signals that regulate the shuttling of HDGF between the nucleus and the cytoplasm remain to be elucidated. Other studies have recently postulated that the cell cycle stage or cellular stress may influence the intracellular localization of HDGF [20,24,25] and mechanistic investigations revealed that p53 repressed HDGF transcription by altering HDAC-dependent chromatin remodeling [26]. Thus, HDGF may be an important autocrine or paracrine factor for Cx cells, and it may shuttle between the cytoplasm and the nucleus, depending on the cell cycle phase and differentiation state.

## 3. Experimental Section 

### 3.1. Materials 

DMSO (dimethyl sulfoxide) and MTT (3-(4,5-dimethylthiazol-2-yl)-2,5-diphenyltetrazolium bromide) were obtained from Sigma (St Louis, MO, USA). Cell culture medium (MEM), fetal bovine serum, antibiotics, sodium pyruvate, trypsin and phosphate-buffered saline (PBS) were purchased from Gibco, BRL (Grand Island, NY, USA). Polyvinylidene fluoride membranes (PVDF) (Millipore Billerica, MA, USA) and molecular weight markers were purchased from Bio-Rad (Hercules, CA, USA). All other reagents and compounds were of analytical grade.

### 3.2. Study Subjects 

Tumor tissue samples were collected from 63 patients with clinical stage Ib to IIa cervical adenocarcinoma, all of whom underwent radical hysterectomy between March 1999 and April 2004, at the Gynecologic Oncology Division of Chang Gung Memorial Hospital, Taiwan. This study was approved by the hospital’s ethics committee. Serum levels of the tumor marker, carcinoembryonic antigen (CEA), were recorded before surgery (Table 1A,B). The clinical stage of the disease was determined according to the 1995 FIGO (International Federation of Gynecology and Obstetrics) staging criteria for cervical cancer. Clinical data, including the patient’s age, stage, histological grade, tumor size at the largest dimension and lymph-vascular space invasion, were obtained from the medical charts and pathological reports. Two co-author of this study, Dr. Shun-Chen Huang and Chao-Cheng Huang, who is a pathologist, performed a pathological slide review. Deep cervical stromal invasion was defined as a tumor invading the outer third of the cervical stroma. Lymph-vascular space invasion was considered to be present when tumor cells were noted within a vascular or lymphatic space lined by flattened endothelial cells. The differentiation of cervical adenocarcinoma was classified into three groups, as well, moderately and poorly differentiated, according to the opinions of the pathologists.

### 3.3. Immunohistochemistry of HDGF in Cervical Adenocarcinoma Clinical Samples 

HDGF was tested by immunohistochemistry in the tumor and non-tumor sections of the clinical samples for comparison and correlation of the expression of HDGF with the histological tumor grade (differentiation) and duration of recurrence of the patients. The cryostat sections of the tissue specimens were mounted on slides, washed twice with TBS and blocked with normal rabbit serum. The sections were incubated with the primary antibodies of each factor followed by 1 h of incubation with the second antibodies with peroxidase conjugate. Antibody staining was visualized with 3,3-diaminobenzidine tetrahydrochloride (DAB, Sigma) in 0.1 M Tris, pH 7.2, containing 0.01% H_2_O_2_. The sections were then counterstained with Harris’s hematoxylin, dehydrated and mounted with mounting medium. The specimens were classified based on immunohistochemical results (Table 1). All immunohistochemically-stained sections were examined in a blinded manner without any knowledge of the clinical pathological variables or the patients’ outcome. Staining of endothelial cells in the non-cancerous areas of each specimen was used as an internal positive control. Constant HDGF expression in the endothelial cells has been reported [19]. The HDGF expression pattern was independently evaluated in the nucleus and cytoplasm; cells showing staining intensity similar to or stronger than that of the endothelial cells in the nucleus or cytoplasm were regarded as nucleus-positive or cytoplasm-positive, respectively.

**Table 1:** Immunohistochemical results. LVSI, lymph-vascular space invasion; LNM, lymph node metastasis; AG, advanced grade.

	HDGF (%)		
Variable	Tumor	Normal	*p* value
LVSI	85.33	45.75	<0.001
LNM	86.83	50.00	<0.001
Recurrence	87.14	58.0	<0.001
AG	88.75	46.87	<0.001

### 3.4. Immunohistochemical Scoring 

The labeling index of cytoplasmic HDGF staining was graded by two pathologists. The staining was stronger than in the positive controls. Samples with >90% of tumor cells were defined as expressing positive immunoreactivity, both in the nucleus and cytoplasm.

### 3.5. Western Blotting 

The tissue samples were excised and frozen immediately at −70 °C. The samples were then homogenized in homogenizing buffer containing a protease inhibitor cocktail. The amount of protein in the lysate was determined using the Bradford method (Bio-Rad, Hercules, CA, USA). Total proteins were separated using 6% or 12% SDS-polyacrylamide gel, transferred onto nitrocellulose membranes (Schleicher and Schnell, Keene, NH, USA) and left at 30 volts overnight. The membranes were then blocked with 5% non-fat milk overnight and incubated with anti-β-actin (1:10,000, Sigma, Saint Louis, MO, USA) and anti-HDGF (1:1000, R&D, Minneapolis, MN, USA) for 2 h. The blots were further washed 3 times for 10 min each time and then incubated with the secondary antibody (1:5000, anti-rabbit or anti-mouse IgG, Santa Cruz, CA, USA) for 1 h. Antigens were visualized using a chemiluminescence kit (ECL, Amersham, Chicago, IL, USA) followed by autoradiography (Hyperfilm, Amersham, Chicago, IL, USA).

### 3.6. Cell Cultures 

Human cervical cancer cells (Hela cells) were purchased from the Food Industry Research and Development Institute (Hsinchu, Taiwan). The human cervical cell lines, SiHa, were purchased from the American Type Culture Collection (Manassas, VA, USA). The primary cell lines (#1 and #2) were provided by Dr. Chou [21,22]. The cells were maintained on culture dishes, in MEM supplemented with 10% (*v/v*) FBS and cultured in an incubator at 37 °C in an atmosphere containing 5% CO_2_.

### 3.7. Cell Proliferation Assay 

Cells were seeded into 96-well culture plates at 10,000 cells/well. Different cell wells were treated with 0, 0.02 and 0.2 nM HDGF for 1 to 2 days. MTT dye (1 mg/mL) was added to each well for an additional 4 hours following treatment. The reaction was stopped by the addition of DMSO, and optical density was measured at 540 nm using a multi-well plate reader (Powerwave XS, Biotek, Winooski, VT, USA). In the absence of cells, background absorbance of the medium was subtracted. The results were expressed as a percentage of the controls, which was considered 100%. Each assay was performed in triplicate, and the results were expressed as the mean (±SEM). 

### 3.8. Adenoviruses Gene Delivery 

The recombinant adenoviruses containing green fluorescent protein (Ad-GFP), 6x-histidine (6xHis)-tagged HDGF cDNA (Ad-HDGF) and specific small-interfering RNA of HDGF (Ad-HDGF RNAi) were generated as previously described [24,25]. The optimal condition for adenovirus vectors to infect oral cancer cells was determined at a multiplicity of infection (MOI) of 200, at which more than 80% of cells expressed as transgenic without overt cytotoxicity.

### 3.9. Statistical Analysis 

SAS software (Statistical Analysis System Institute, Cary, NC, USA) was used for all statistical analyses. The χ^2^ test and Fisher’s exact probability test were used to examine the relationship between HDGF expression and clinicopathological variables of prognosis. Multivariate analysis of the factors related to survival was analyzed by a Cox proportional hazards regression model. A *p*-value of less than 0.05 was considered to be statistically significant.

## 4. Conclusions 

In summary, we present evidence of an elevated HDGF expression in Cx specimens. Furthermore, we showed that increased HDGF levels are correlated with the progression of Cx carcinogenesis and that they can predict a poor prognosis for patients with Cx after surgery. Our findings indicate that HDGF may be a candidate gene for the development of diagnostic and therapeutic strategies for Cx.

## Supplementary Materials

Supplementary materials can be found at http://www.mdpi.com/1422-0067/15/11/21492/s1.
